# Self-Adjustment Energy Efficient Redeployment Protocol for Underwater Sensor Networks

**DOI:** 10.3390/s23208514

**Published:** 2023-10-17

**Authors:** Saoucene Mahfoudh

**Affiliations:** School of Engineering, Computing, and Design, Dar Al-Hekma University, Jeddah 6702, Saudi Arabia; smahfoudh@dah.edu.sa

**Keywords:** underwater sensor network, network deployment, coverage, energy efficiency, water currents

## Abstract

The diversity of applications supported by Underwater Sensor Networks (UWSNs) explains the success of this type of network and the increasing interest in exploiting and monitoring seas and oceans. One of the most important research fields is network deployment, since this deployment will affect all other research aspects in the UWSNs. Moreover, the initial random deployment resulting from scattering underwater sensor nodes on the network area’s surface does not ensure this area’s coverage and network connectivity. In this research, we propose a self-adjustment redeployment protocol that enhances network coverage and connectivity while reducing the energy consumed during network deployment. This protocol takes into account the peculiar dynamism of the underwater environment due to the water currents. First, we study the impact of these water currents on network deployment. Then, we exploit these water currents to adjust the nodes’ positions to achieve total area coverage and reduce the energy consumed during the deployment by reducing the total distance traveled by the underwater sensor nodes. Simulation results show that the proposed protocol achieves a very high coverage rate (97%) and reduces the distance traveled by nodes during the deployment by 41%.

## 1. Introduction

Underwater Sensor Networks (UWSNs) constitute an emergent technology that has caught the interest of many researchers in the last few years. The diversity of applications supported by the UWSNs explains the success of this type of network. These networks serve a multitude of purposes, ranging from the study of marine ecosystems and climate models to supporting offshore industries like oil and gas exploration, maritime security, and underwater infrastructure maintenance [1,2]. To enable network communication in the harsh underwater environment, underwater sensors mostly employ acoustic signals using acoustic modems [3,4]. However, acoustic signals still have many challenges and difficulties, including high propagation delay, high attenuation, and limited bandwidth [5]. Furthermore, the Doppler effect is another problem due to the movement of the sender and the receiver caused by the water currents, which affects the signal intensity [6,7]. Much research focuses on solutions for UWSNs, such as network deployment, energy efficiency, network connectivity, routing protocols, etc., while taking into consideration the different characteristics and constraints of the UWSNs [8].

In this work, we are interested in network deployment, which is a critical step to achieve the application objective since it affects all other UWSN aspects. In fact, the UWSN is composed of interconnected underwater sensors deployed in the underwater aquatic area. The objective of these sensors is to collaboratively monitor and gather information in the network area according to the requirements of the network application. The collected data are then sent to a surface sink node on a multi-hop path [8]. However, the initial random deployment is not efficient: it does not ensure either the connectivity or the coverage of the network area that needs to be monitored.

Many deployment solutions [9] based on the swarm [10], graph coloring [11], virtual forces [12], etc., are proposed for the deployment of the underwater sensor networks. Most of these solutions suppose an ideal environment in which the sensor can move from one position to another without accounting for external forces and the effect of these forces on the movement of the sensor, its energy, and its final position. One of the most important external forces exerted on these sensors is the water currents. In fact, for the underwater sensor network, the water currents directly affect the network deployment. Our target is to propose a redeployment protocol that enhances network coverage and connectivity and takes into consideration the water currents in the underwater environment.

On the other hand, the energy constraint in underwater sensors, manifested by the limited amount of energy in the sensors’ batteries and the difficulty in replacing or recharging these batteries [13], is considered one of the most challenging points when designing solutions for UWSNs. For these reasons, the proposed solution should take into consideration the energy constraints in the UWSN and reduce the energy consumed during the network deployment.

Our contributions in this research work are:

1. Study the impact of the water currents on the deployment protocol.
2. Propose a solution to achieve the deployment while considering the water currents.
3. Exploit these currents to reduce the energy consumed during the deployment.
4. Evaluate the performance of the proposed protocol using a network simulator.

Even though our solution can be applied to any proposed deployment protocol, in this research, we chose the distributed virtual forces algorithm (DVFA) [14] for our discussion.

The rest of the paper is organized as follows: Section 2 summarizes the research related to the focus of this paper. Section 3 describes the assumptions of our solution. Section 4 presents the distributed virtual forces algorithm used as the deployment protocol reference in this paper. Section 5 discusses the impact of the water currents’ force on the network deployment. Section 6 presents the self-adjustment deployment protocol, our solution to consider and profit from the water currents during the network deployment. Section 7 and Section 8 present the simulation details and the performance evaluation of the proposed solution, respectively. Finally, Section 9 concludes this paper.

## 2. Literature Review

The principal task of an UWSN is to ensure coverage of the network area to perform the measurement or monitoring needed by the application. In addition to the coverage, the connectivity is mandatory to deliver all collected data to the sink node [15]. However, the initial UWSN deployment is usually a random deployment that ensures neither coverage nor connectivity. That is why much research has focused on UWSN redeployment, and many redeployment techniques are proposed [16]. These techniques can be classified into three categories based on node mobility: static deployment, depth adjustment deployment, and free mobility deployment.

- In static deployment [17,18,19], the underwater sensor nodes are manually deployed in prefixed positions calculated to ensure area coverage and network connectivity. Then, these nodes cannot adjust or change their positions. These solutions need knowledge of the monitored area, which is not practical in an underwater environment where the water currents and wind (in the case of 2D ocean surface deployment) change the shape of the topology frequently.
- In depth adjustment deployment, the underwater sensor nodes can only move vertically and change their depths by using a tether attached to anchors at the surface of the ocean or via a mechanism described in [20]. The target of these solutions is to reduce the coverage overlaps in the first random deployment and, hence, increase the network area coverage [11,21,22,23].
- In the last category, free mobility deployment, the underwater sensor nodes can move freely in any direction by following the deployment algorithm. In this paper, we propose a deployment solution for networks with free-movement sensors. In the following, we present some related work in this category.

### Free Mobility Deployment

In recent ye ars, much research has exploited the capacity of the underwater sensor nodes to move freely in the underwater environment to propose a deployment protocol for UWSNs.

Xia et al., in [10], propose a particle swarm-inspired underwater sensor self-deployment (PSSD) algorithm. The aim of this algorithm is to relocate sensor nodes to efficiently cover the network area. This algorithm simulates the flying behavior of bird flocking or fish schooling to cover the area with a high density of events and make the distribution of sensors (particles) match those events. However, this solution considers only the coverage problem without considering network connectivity or energy conception in the UWSN. This solution is extended in [24] by Wang et al. The authors propose a distributed hybrid fish swarm optimization algorithm (DHFSOA) based on the operation of an artificial fish swarm system to improve the coverage efficacy of the event set and to avoid blind movements of sensor nodes. Simulations show that the DHFSOA, compared with PSSD, can maintain higher event coverage and coverage efficacy of event sets, and reduce the total nodes moving during network deployment. However, these studies do not take into consideration the water currents during the sensor node displacement.

In [25], Jiang et al. propose a deployment algorithm for a UWSN based on a connected tree. This connected tree is built by the sink node to ensure network connectivity. To reduce the coverage overlaps and increase the coverage rate of the network, the distance between parents and child nodes is then adjusted.

In [26], a deployment algorithm based on a building-connected dominating set is proposed. The target of this solution is to ensure connectivity with the sink while maximizing the coverage rate. To achieve this, the connected dominating set is built by the sink node to ensure full connectivity in the network. Then, the sink node optimizes the nodes’ deployment by adjusting the nodes’ location. The proposed solution is a centralized solution based on broadcast messages, which is costly from an energy and bandwidth point of view. Moreover, this algorithm does not consider the characteristics of the underwater environment, like the water currents that affect the node’s displacement and position.

In [27], the authors propose a Fully Distributed Deployment Algorithm to construct 3D multi-level k-barrier coverage for a UWSN using available sensor nodes. The target is to ensure the security of the network area and detect any intrusion. The idea is first to construct the one-barrier coverage with the minimum number of sensor nodes. Then, based on this construction and the final position of sensor nodes, a 3D maximum-level k-barrier coverage solution with all sensor nodes is proposed. However, the proposed solution does not take into consideration the effect of the water currents in the final deployment and the sensor nodes’ displacements.

In [12], an adaptation of the distributed virtual force algorithm to the underwater environment is proposed. The main objective of this research is to achieve full coverage of the network area while minimizing the number of nodes used. The idea of the algorithm is to maintain a predetermined distance threshold between neighboring nodes. Hence, if the distance between two neighboring sensors is smaller than the threshold, a repulsive force will be exerted to avoid node stacking. On the other hand, if the distance between two neighbor nodes is higher than the threshold, an attractive force will be exerted to avoid the coverage hole. To determine the distance threshold, a regular dodecahedron is used to fill the network area where the sensor node will be located at the center of the dodecahedron, and it will have 12 neighbors with the same distance. The water currents are added as a force in the virtual force equation. Simulation results show that the proposed solution ensures a very high coverage rate. However, the impact of the water currents on the deployment protocol is not shown.

In [28], Fattah et al. propose a hybrid multi-objective node deployment for the energy-coverage problem in mobile underwater sensor networks. The primary objective of this research is to propose a novel approach for deploying sensor nodes in mobile underwater environments that can simultaneously optimize energy consumption and coverage performance. The research generates a set of Pareto front solutions, representing a range of trade-offs between energy efficiency and coverage. The Pareto front solutions empower decision makers to effectively monitor the region of interest. Simulation results show that the proposed solution is more effective than the current multi-objective algorithms.

Table 1 summarizes and compares the solutions presented in free movement deployment solutions for UWSNs in terms of coverage, connectivity, energy efficiency, and water currents consideration. We notice that the first objective of all this research is area coverage, which is very important to achieve the application target. However, the water currents are almost never taken into account in the network deployment and nodes’ displacements.

In this work, we first study the impact of the water currents on the deployment protocol. Then, we propose a solution to achieve the deployment, considering the water currents. Even though our solution can be applied to any proposed deployment protocol, in this paper, we chose the distributed virtual forces algorithm (DVFA) for our discussion. In the next section, we briefly explain the assumptions of our solution.

## 3. Assumptions

In this research, we suppose that each sensor node in the UWSN has the ability to sense, communicate, and change its position by moving freely in the 3D network area [6,7]. For underwater communication, acoustic modems are used to communicate between underwater sensors and between underwater sensors and the surface stations [3,4]. The sensing capability of the underwater sensors depends on the application target, and the sensing technologies can include sonar, cameras, etc. For the underwater displacement, we suppose that for each sensor, an underwater robot serves as a vehicle that carries this sensor and enables its deployment in the network area. Recent research is focused on the development and enhancement of underwater robots’ movement capabilities in the underwater environment, as presented in [29,30]. Each sensor node has a sensing range $R_{s}$ and a transmission range $R_{t}$.

We assume that the UWSN is composed of *n* underwater sensor nodes scattered randomly on the surface of the monitored area. The sensor node set is *S*, where *S* = {$s_{1}$,$s_{2}$,…,$s_{n}$}.

The UWSN area is 100% covered if any point in the 3D network area is at least covered by one sensor node. A point *P* in the network area with the coordinate (x,y,z) is said to be covered, if there exists a sensor node $s_{i}$ with the coordinate $\left(x_{i},y_{i},z_{i}\right)$ such that $d\left(P,s_{i}\right)<R_{s}$, where $d(P,s_{i})$ is the Euclidean distance between *P* and $s_{i}$.

Moreover, we suppose that $R_{t}\geq \sqrt{3}R_{s}$ since it is proven that when the above relation between $R_{s}$ and $R_{t}$ is applied, the connectivity will be implied if the coverage is achieved. Thus, if network coverage is ensured, network connectivity will also be ensured.

## 4. Description of 3D Distributed Virtual Forces Algorithm (3D-DVFA)

The target of the 3D-DVFA [14] is to relocate sensors in the network area uniformly while maintaining a calculated target distance, $D_{th}$, between neighbor nodes. In each iteration, each sensor will calculate its new position based on localized information. This information is about its position and the position of its sensor neighbors. The new position of a sensor node is calculated according to the sum of the forces exerted on it by its neighbors.

An iteration is defined as follows:

1. Step 1: Each node broadcasts a Hello-message each Hello-period to discover its neighbors.
2. Step 2: Each node computes the virtual forces exerted on it by its neighbors.
3. Step 3: Each node determines its new position by computing the resultant force. At each iteration, the algorithm computes for any neighbor sensor $s_{i}$ located at $\left(x_{i},y_{i},z_{i}\right)$, the force denoted $\vec{F_{ij}}$, exerted by any sensor $s_{j}$ located at $\left(x_{j},y_{j},z_{j}\right)$. Let $d_{ij}$ be the Euclidean distance between $s_{i}$ and $s_{j}$ and $D_{th}$ the deployment target distance.
  (1) $$\vec{F_{ij}}=\left\{\begin{array}{cc} K_{a}\left(d_{ij}-D_{th}\right)\frac{x_{j}-x_{i},y_{j}-y_{i},z_{i}-z_{j}}{d_{ij}} & \mathrm{if}\;d_{ij}<D_{th}\\ K_{r}\left(D_{th}-d_{ij}\right)\frac{x_{j}-x_{i},y_{j}-y_{i}-z_{j}-z_{i}}{d_{ij}} & \mathrm{if}\;d_{ij}>D_{th}\\ Null & \mathrm{if}\;d_{ij}=D_{th} \end{array}\right.$$
  where $K_{a}$ is a positive attractive coefficient and $K_{r}$ is a positive repulsive coefficient. These coefficients are used to tune the attractive and repulsive forces. The resulting force exerted on the sensor $s_{i}$ is $\vec{F_{i}}$
  (2) $$\vec{F_{i}}=\sum_{j}\vec{F_{ij}}$$
  where *j* represents the sensor $s_{i}$’s neighbors.
  The new position of the node $s_{i}$ located at position $\left(x_{i},y_{i},z_{i}\right)$ will be $\left(x_{i}^{\prime }=x_{i}+F_{ix},y_{i}^{\prime }=y_{i}+F_{iy},z_{i}^{\prime }=z_{i}+F_{iz}\right)$.
4. Step 4: The node moves to its new position.

Each iteration has a duration of one Hello period. The target of this algorithm is to spread sensor nodes in the whole area while ensuring network connectivity and area coverage. The result of the sensor network deployment using 3D-DFVA is very satisfactory with a good coverage rate and uniform density.

## 5. Impact of the Water Currents on the Network Deployment

The dynamic nature of the aquatic environment leads to a continuous change in the network topology. This is due to the water currents and the surface wind [31]. For the underwater sensor network, the water currents directly affect the network deployment. These water currents, in the realistic case, change with the depth. Many research works consider network redeployment without taking into consideration the water currents. In this section, we will show the difference between considering the ideal case in which there are no water currents and the real resulting network deployment with water currents.

**Figure 1:**
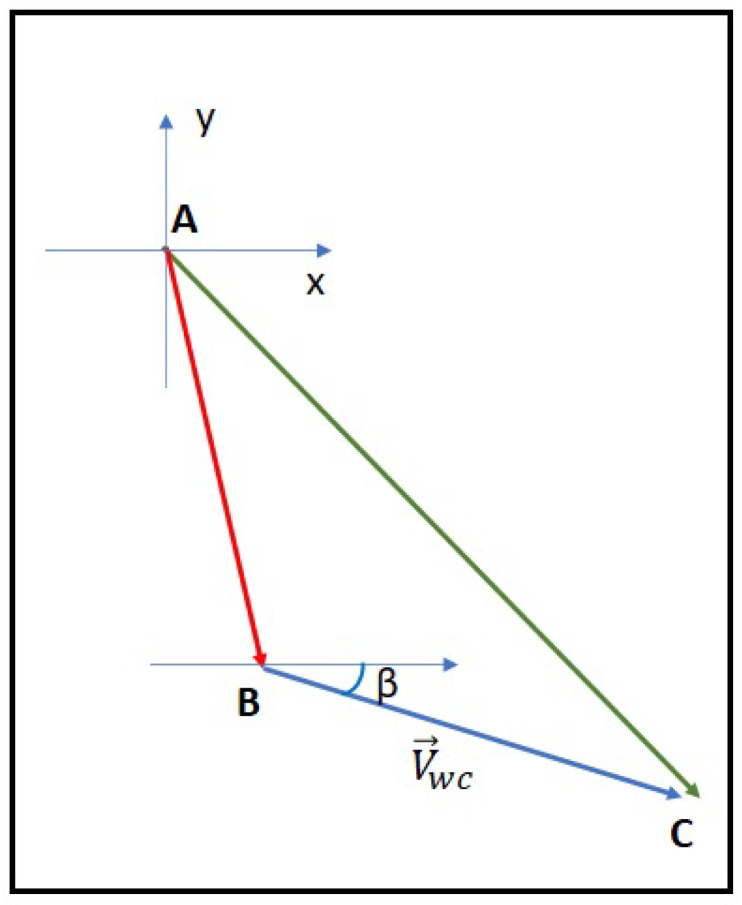
Real displacement in the presence of water currents.

Figure 1 shows the effect of the water currents on the node displacement. We suppose the sensor $s_{i}$ in position *A*, and its new position calculated by the deployment algorithm is *B*. Hence, the sensor $s_{i}$ should move from *A* to *B*. However, when the sensor starts moving and takes the direction to reach *B*, due to the water currents, this direction will deviate, and the sensor will reach position *C* instead.

By simulation, we show the difference between the deployment protocol using 3D-DVFA in an ideal environment without water currents and the real deployment resulting with water currents. For the simulation, we used the simulator Aquasim, the underwater network simulator based on network simulator ns2. The number of sensors is 400 nodes deployed initially randomly at the surface of the ocean in a 3-D area. The speed of the sensor is 2.5 m/s and the speed of the water currents depends on the depth. The simulation parameters are presented in Section 7.2.

**Figure 2:**
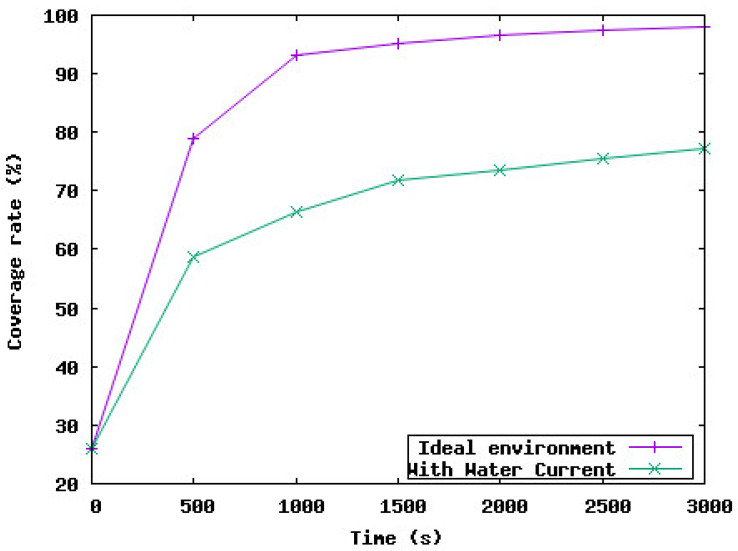
Coverage rate.

**Figure 3:**
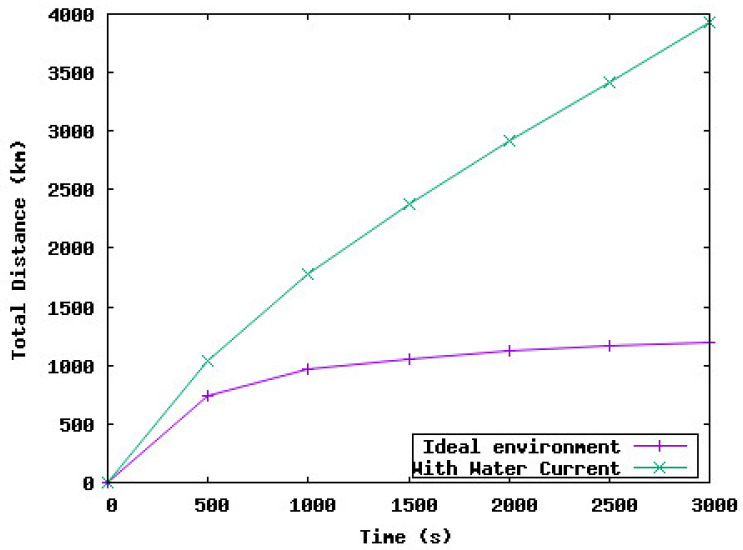
Total distance traveled by the underwater sensor nodes.

Figure 2 shows the coverage rate achieved by the deployment of the UWSN with and without considering the water currents. Based on these results, we notice that when the deployment protocol shows the coverage rate is 98%, the real network coverage does not exceed 78%. This rate is unsatisfactory from the application point of view which aims to maximize the area monitoring and so the coverage rate. Moreover, the total distance traveled by sensor nodes during the network deployment is 5 times higher than the theoretical distance traveled by sensor nodes as shown in Figure 3.

Based on these results, we can conclude that the water currents heavily affect the network deployment. Hence, these water currents should be considered in the deployment protocol. Moreover, the water currents can impact the energy consumed during the deployment. In fact, if the movement towards the new position is in the same direction as the water currents, in this case, the sensor will consume less energy to reach its position. However, if the movement to the new position is against the water currents, the node will expend more energy to reach its new position.

In the next section, we will extend the 3D-DVFA protocol to take into account the water currents and study the effect of the water currents on the energy consumed during the deployment. Our new protocol is called Self-Adjustment DVFA (SA-DVFA).

## 6. Self-Adjustment Deployment Protocol

The water currents influence the route of the underwater sensor node. It will affect the speed of the sensor as well as its trajectory. It must, therefore, be anticipated to reach the destination. Our protocol is a self-adjustment protocol because each node after the new destination is calculated by the 3D-DVFA protocol will calculate its new position, direction, and speed to reach the precalculated destination. This new position takes into account the speed and direction of the water currents. In the following, we first describe the proposed deployment protocol. Then, we explain how we can exploit the water currents to reduce the energy consumed during the deployment.

### 6.1. Self-Adjustment Deployment Protocol Description

**Figure 4:**
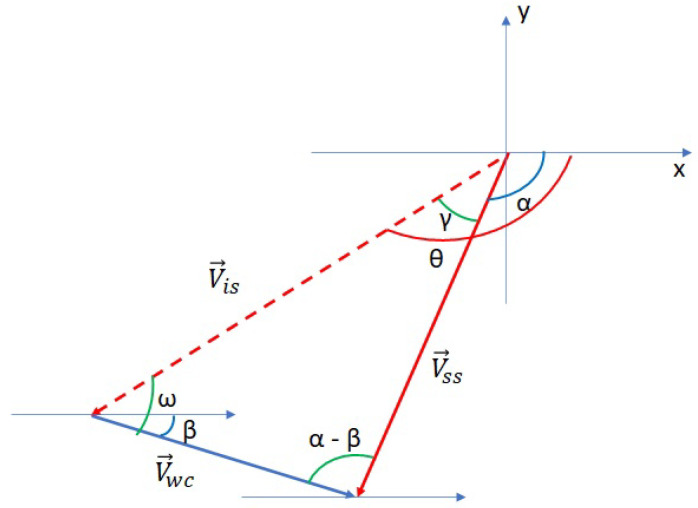
Water currents impact on the sensor displacement.

In Figure 4, we show the difference between the calculated new position which is *B* (calculated with the 3D-DVFA algorithm), and the direction and distance that should be traveled to reach the position *B* considering water currents. The idea is the same idea of the boat navigation used to reach a specific destination. In fact, based on the speed and the direction of the water currents, a new direction is calculated to reach the destination. Hence, the trajectory is not a line matching the starting point *A* and the arriving point *B*. Similarly, in the sensor network, in each deployment algorithm iteration, the sensor will define its direction and the distance traveled based on the position calculated by the 3D-DVFA algorithm and the water currents and its direction.

The new direction of the sensor node is defined by the angle θ. As shown in Figure 4, we have

(3) $$\vec{V_{ss}}=\vec{V_{is}}+\vec{V_{wc}}$$

where $\vec{V_{ss}}$ is the sensor arrival speed, $\vec{V_{is}}$ is the the sensor initial speed, and $\vec{V_{wc}}$ is the water currents speed.

To determine the new direction, θ, we use the sine rule:

(4) $$\frac{V_{ss}}{\sin\omega }=\frac{V_{wc}}{\sin\gamma }=\frac{V_{is}}{\sin(\alpha -\beta )}$$

(5) $$\sin\gamma =\frac{V_{wc}\sin\left(\alpha -\beta \right)}{V_{is}}$$

(6) $$\gamma =\sin^{-1}\frac{V_{wc}\sin\left(\alpha -\beta \right)}{V_{is}}$$

(7) θ=γ+α

where β is the water direction, α is given by the 3D-DVFA algorithm based on the calculated new position of the sensor node. Hence, to reach its calculated new position, the sensor node will move using the new direction θ and its speed $V_{is}$.

### 6.2. Energy Efficient Deployment

In underwater sensor networks, the energy constraint represents a critical challenge that directly impacts network performance and lifetime. This energy constraint is manifested by the limited capacity of sensors’ batteries and the difficulty in replacing or recharging these batteries [13]. There are two aspects of energy consumption in the UWSN: The first is related to the sensor movement, and the second is related to the communication between sensors (energy consumed during the transmission and reception of messages). The energy consumed in the communication between nodes in our protocol is the energy consumed by the Hello message, which is a periodic message sent at the beginning of each iteration, as explained in Section 4. Hence, the number of Hello messages is fixed, and so the energy consumed by this message cannot be reduced at this level. Therefore, in this work, we focus on the energy consumed by the node displacement during the deployment. In fact, large displacement costs too much energy [32]. Our target is to reduce this energy as much as possible.

**Figure 5:**
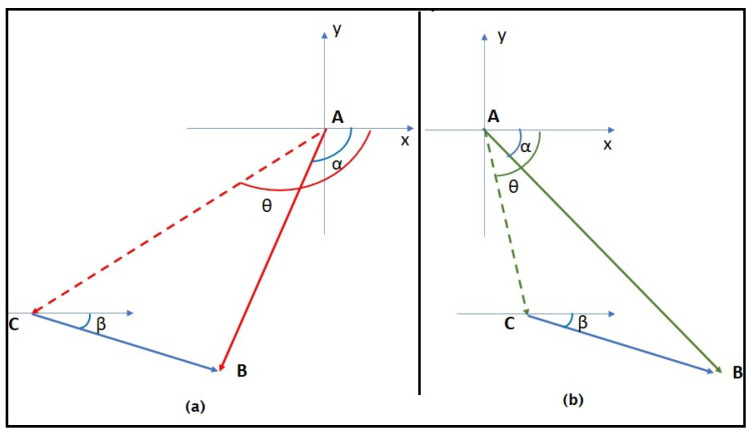
(**a**) Water currents impact when the sensor displacement is in the opposed direction, (**b**) Water currents impact when the sensor displacement is in the same direction.

As explained above, the water currents have a speed and a direction. When the sensor moves from one position to another, these water currents based on its direction and the direction of the displacement of the sensor can help (Figure 5b) or resist (Figure 5a) to this movement. Clearly, if the displacement of the sensor is with water currents, the sensor will consume less energy to reach its destination since it is helped by the water currents. However, if the displacement of the sensor is against the water currents, the sensor will consume more energy to reach its destination. Our target in this work is to profit from these water currents to reduce the energy consumed without reducing the performance of our deployment protocol. Hence, we aim to reduce, as much as possible, the displacement of the sensor if it is against the water currents. To achieve this, we will calculate the work of the movement of the sensor from *A* to *B*. Then, if the work *w* is positive, this means the sensor saves energy in this displacement. However, if the work is negative, this means the sensor dissipates more energy in this displacement.

The node calculates the work of the new movement based on Formula (8):

(8) $$w=\frac{1}{2}m\left(V_{ss}^{2}-V_{is}^{2}\right)$$

Based on Formula (4), we have:

(9) $$V_{ss}=\frac{V_{is}\sin\omega }{\sin(\alpha -\beta )}$$

(10) ω=π−γ−(α−β)

(11) $$V_{ss}=\frac{V_{is}\sin\left(\gamma +\left(\alpha -\beta \right)\right)}{\sin(\alpha -\beta )}$$

While γ is calculated by Formula (6).

To summarize, our solution, SA-DVFA, consists of three parts:

1. Each sensor node calculates its new position based on the 3D-DVFA algorithm.
2. Then, based on the water currents, the sensor calculates the new direction to reach its position.
3. Before moving in this direction, the sensor calculates the work of this displacement. If the work is positive, the sensor node moves immediately to its new position. If the work is negative, the sensor waits the next iteration hoping that the neighborhood will change. If after *k* times the work remains negative, the sensor moves to its new position.

We can calculate the upper and lower boundaries of the work. For this target, we use the cosine rule on the $V_{ss}$ shown in Figure 4:

(12) $$V_{ss}^{2}=V_{is}^{2}+V_{wc}^{2}-2V_{is}V_{wc}\cos\left(\omega \right)$$

if we replace $V_{ss}$ in Formula (8), we obtain:

(13) $$w=\frac{1}{2}mV_{wc}\left(V_{wc}-2V_{is}\cos\left(\omega \right)\right)$$

- The minimum work is obtained as the following
  (14) $$min\left(w\right)=min\left(V_{wc}-2V_{is}\cos\left(\omega \right)\right)=max\left(2V_{is}\cos\left(\omega \right)\right)$$
  and
  (15) $$max\left(2V_{is}\cos\left(\omega \right)\right)=max\left(\cos\left(\omega \right)\right)$$
  Hence, the minimum work is obtained when cos(ω)=1, so ω=0.
- Similarly, the maximum work is obtained with min(cos(ω)), so cos(ω)=−1 and ω=π.

**Figure 6:**
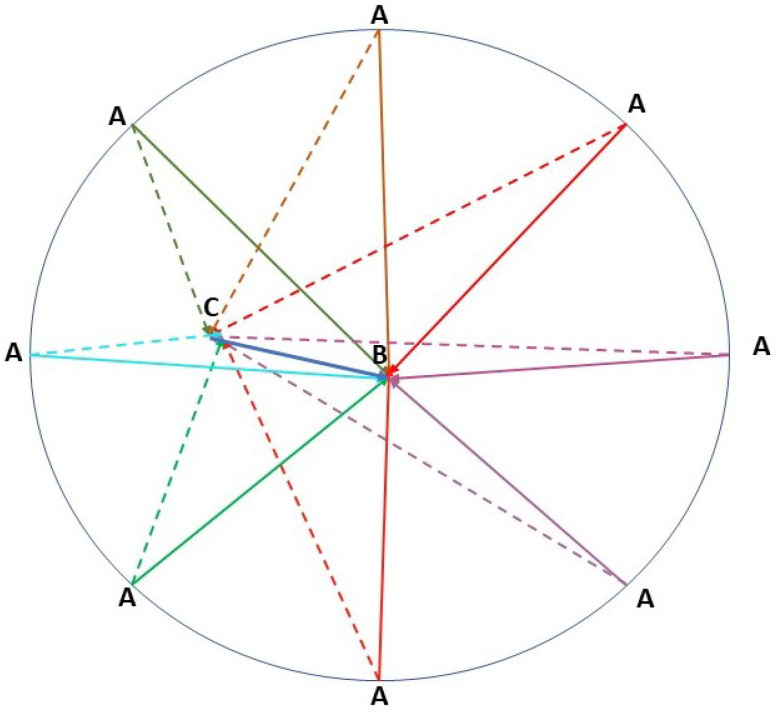
Water currents impact on the sensor displacement: the difference between the movement with the currents and against the currents.

Figure 6 shows the difference between the sensor movement with the currents and against the currents. The solid lines represent the direction and destination of the node *A* calculated by the deployment protocol (3D-DVFA). However, the dashed lines represent the new direction that the node should follow to reach the final position *B*. This new direction is calculated by the self-adjustment protocol (SA-DVFA) taking into account the water currents. If the distance AC<AB, the sensor node will save energy in this displacement. Nevertheless, if the distance AC>AB, the sensor node will consume more energy with this displacement.

In the next step, we will study the performance of our solution by simulation.

## 7. Simulation Details

In this section, we first present the simulation tool used to evaluate the performance of our proposed protocol. Then, we describe the simulation environment.

### 7.1. Simulation Tool

To evaluate the performance of our solution, we used the network simulator Aquasim [33,34], which is a network simulator for underwater sensor networks implemented based on the network simulator ns2. Each simulation result is the average of 20 simulations with different initial random network deployments on the surface of the ocean in the 3-D network area. In the following subsection, we describe the simulation environment.

### 7.2. Simulation Environment

As explained in [31], the water currents velocity is a horizontal force that changes with the depth. In our simulation, we considered the example of the Gulf Stream which is an intense, warm ocean current in the western North Atlantic Ocean. It moves north along the coast of Florida and then turns eastward off of North Carolina, flowing northeast across the Atlantic. The velocity of the Gulf Stream current is fastest near the surface. Its average speed is 6.4 km per hour, and its maximum is 9 km per hour [12,35]. Based on this, we varied the water currents with the depth from 2 m/s to 0.5 m/s, and β the direction of the currents is equal to 0.

All simulation parameters are presented in Table 2.

## 8. Performance Evaluation

Our target in this section is first to show the self-adjustment deployment algorithm’s performance in terms of coverage rate and then to study the effect of the water currents on the energy dissipated during the deployment phase.

### 8.1. The Coverage Rate

**Figure 7:**
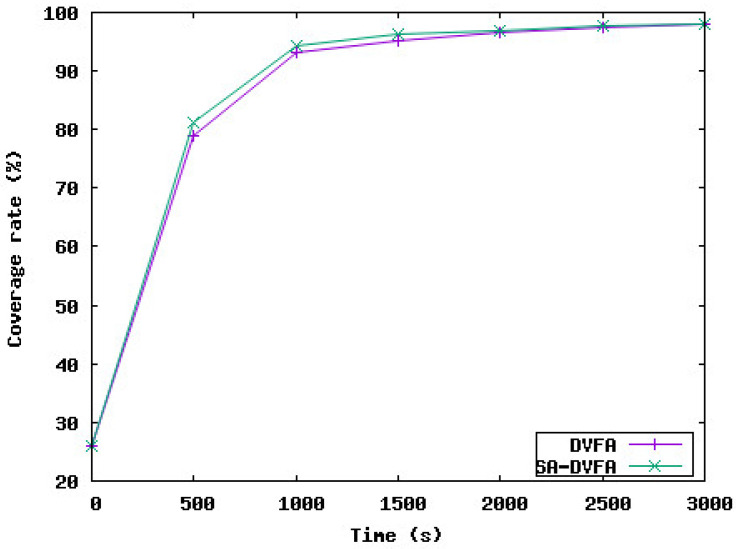
Comparison of the coverage rate between DVFA and SA-DVFA.

Figure 7 compares the coverage rate achieved with the 3D-DVFA protocol without taking into consideration the water currents and the proposed protocol SA-DVFA. The target is to study the effect of considering the water currents in one of the most important sensor network performance parameters, which is the coverage rate. Based on the simulation results, we notice that the coverage rate achieved by our protocol is 98%, the same coverage rate achieved by 3D-DVFA which is a very acceptable rate from the application point of view. In addition, considering the water currents in our protocol does not affect the time needed for the underwater sensor nodes to be deployed in the network area. This shows that our protocol did not reduce the good performance achieved by the 3D-DVFA protocol. Indeed, SA-DVFA takes into account the water currents to readjust the direction of the sensor node displacement to reach the final destination calculated by the 3D-DVFA algorithm.

### 8.2. Energy Efficiency Evaluation

To evaluate the energy consumed during the deployment phase, we measured the distance traveled by sensor nodes to reach their final position in the network area. Our target is to reduce the sensor nodes’ displacements as much as possible during the deployment without affecting the coverage rate.

**Figure 8:**
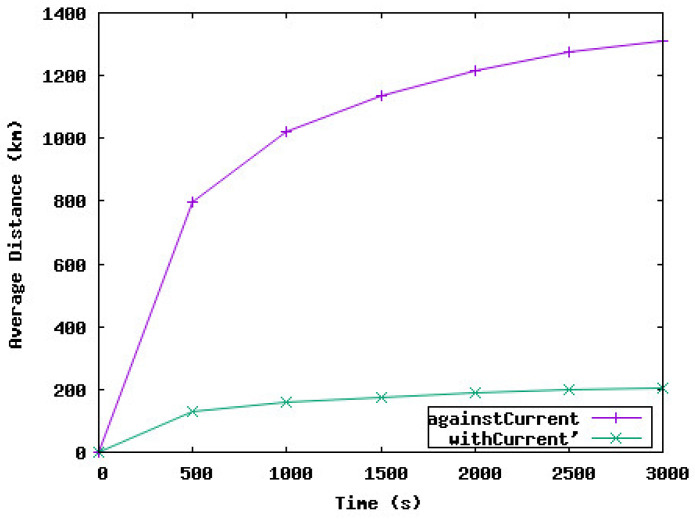
Total distance traveled by the underwater sensor nodes against and with the water currents.

Figure 8 shows the total distance traveled by sensor nodes with and against water currents during the deployment phase. We notice that the total distance traveled by nodes against the currents is much higher than the distance traveled by nodes with the current. Hence, a large amount of energy of the sensor nodes is dissipated in these displacements. Our target is to reduce this energy (and hence the distance traveled by nodes) while maintaining a satisfactory coverage rate. Our solution, SA-DVFA, as explained in Section 6.2, consists of reducing the displacement of nodes if the work is negative because the node will consume more energy to reach its destination.

In the following, we study the effect of delaying some node displacements if the work is negative on the network performance in terms of coverage rate, the time needed to reach this rate, and the distance traveled by nodes during the deployment. *k* represents the number of consecutive times a node must wait if the work is negative. In the following simulation series, we varied *k* from 0 to 5. k=0 means the node should move immediately to the new position and should not wait, even if the work is negative. k=i means the node can wait maximum *i* iteration(s) if the work is negative.

**Figure 9:**
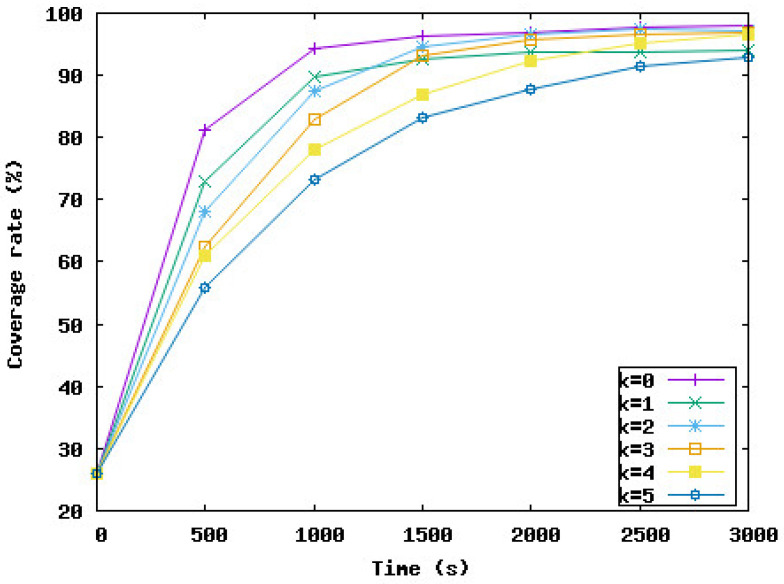
Coverage rate with different values of *k*.

**Figure 10:**
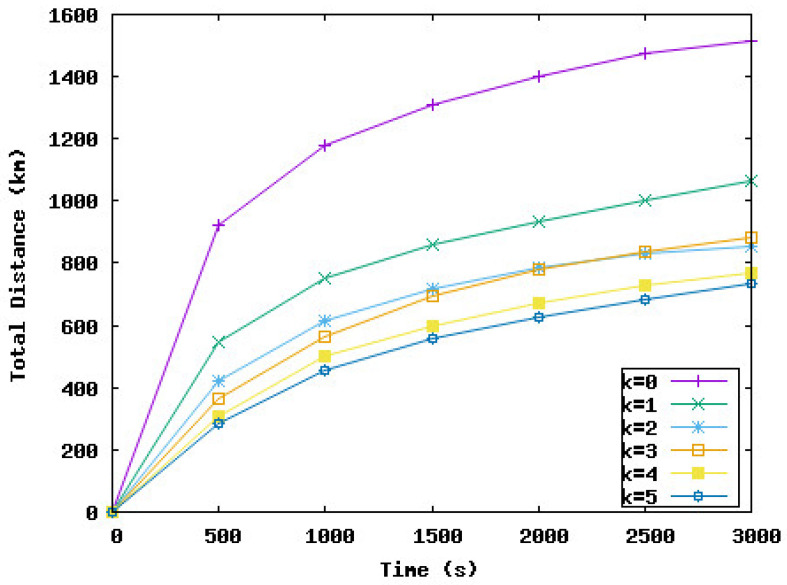
Total distance traveled by the underwater sensor nodes against the water currents with different values of *k*.

**Figure 11:**
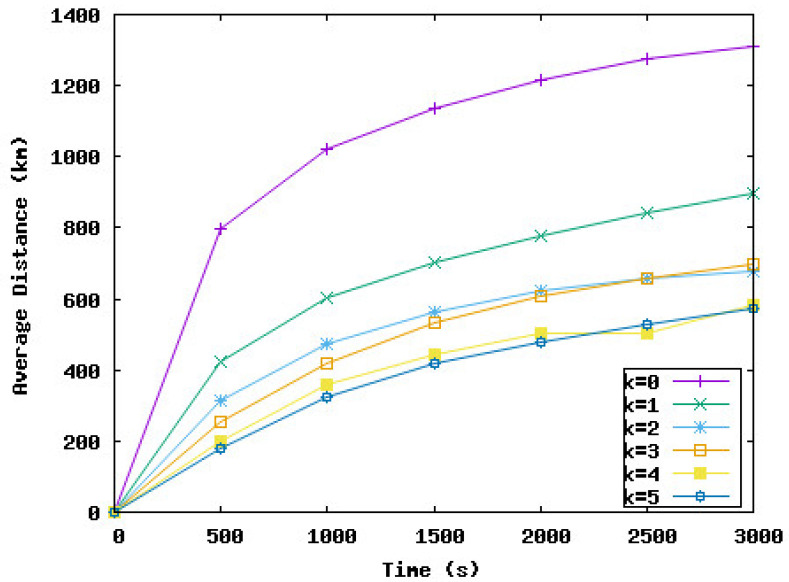
Average distance traveled by the underwater sensor nodes against the water currents with different values of *k*.

**Figure 12:**
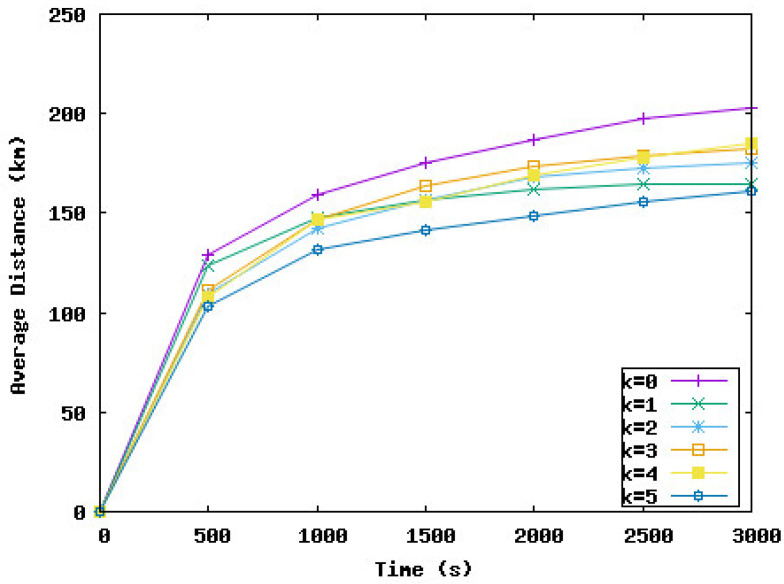
Average distance traveled by the underwater sensor nodes with the water currents with different values of *k*.

Figure 9, Figure 10, Figure 11 and Figure 12 present the coverage rate, total distance traveled by the underwater sensor nodes against the water currents, average distance traveled by the underwater sensor nodes against the water currents, and average distance traveled by the underwater sensor nodes with the water currents, respectively, while varying *k* from 0 to 5.

We remark that the distance traveled by nodes decreases when the value of *k* increases. However, the performance of the network in terms of coverage rate also decreases with the value of *k*. Hence, the target is to obtain a trade-off between the energy consumed and the minimum coverage rate tolerated by the application. For example, if k=3, the coverage rate is reduced by 1%, and this rate is obtained after 2000 s from the beginning of the simulation. However, the distance traveled against the currents and hence the total distance is considerably reduced by 46% and 41%, respectively. The loss of 1% of the coverage can be accepted by the application considering the energy that will be saved. For k=5, the distance traveled by nodes is reduced by 51%. However, the coverage rate decreased from 98% to 92% and this rate was reached at the end of the simulation time. To summarize, the SA-DVFA protocol maintains almost the same good performance as the 3D-DVFA protocol while taking into account the water currents. Moreover, SA-DVFA profits from these water currents to save the underwater sensor nodes by reducing the nodes’ displacements during the deployment phase.

Based on the simulation results, we recommend the value 3 for *k* which means nodes can wait until 3 times if the displacement is against the water currents. With this value, we obtained an accepted coverage rate while decreasing considerably the energy dissipated by the nodes’ displacements during the network deployment phase.

## 9. Conclusions

Given the large number of underwater sensor network applications and the increasing interest in exploiting and monitoring seas and oceans, much new research has focused on this type of network. One of the most important research fields is network deployment. The target of the deployment solutions is to increase area coverage, which is usually not ensured by the initial random network deployment. In addition, maintaining network connectivity and minimizing energy consumption as much as possible were also the objectives of any deployment protocol.

One of the main characteristics of the underwater environment is the dynamism of this environment due to the water currents. In this research, we first studied the impact of these water currents on the deployment protocol. We showed that if this impact is not considered during the design of the protocol, the results of this protocol in a real environment will be different from the simulated results. Then, we proposed a self-adjustment deployment protocol that takes into account the water currents during the deployment. The idea of the proposed protocol is the same as that used by boat navigation to adjust their direction to reach the target destination in the presence of water currents. In the second step, we proposed a solution to reduce the energy consumed during the deployment by reducing the distance traveled by nodes to reach their last positions. The idea is to take advantage of the water currents by favoring the node displacements in the direction of the water currents and avoiding as much as possible displacements against the current. Simulation results show that the proposed solution reduces by 41% the distance traveled by sensor nodes while maintaining a very good coverage rate of 97%.

In future work, we will consider a more realistic underwater environment by introducing obstacles in the network area. Moreover, we will study the non-uniform deployment in which redundant nodes are considered near the sink to balance the load and hence avoid the quick energy depletion of these nodes.

## Figures and Tables

**Table 1:** Comparison of state-of-the-art Free Mobility Deployment solutions in UWSNs.

Reference	Algorithm	Coverage	Connectivity	Energy Efficient	Water Currents
[10]	Practical Swarm	✓	-	-	-
[12]	Distributed virtual force algorithm	✓	✓	Number of messages sent and received	Added as a force in the deployment protocol
[24]	Distributed hybrid fish swarm optimization	✓	✓	Reduce the total node moving distance during node deployment	-
[25]	Connected Tree	✓	✓	Communication energy and node displacement energy consumption	Considered only for the connectivity but not in the deployment protocol
[26]	Connected dominating set	✓	✓	-	-
[27]	Fully Distributed Deployment Algorithm	✓	✓	Reduce sensors displacement by moving to the closest vacant position	Redundant sensors to resolve the water currents coverage and connectivity holes but not considered in the deployment protocol
[28]	Hybrid multi-objective node deployment f	✓	-	Reduce the energy consumed for mobility, sensing, and data communication processes	-

**Table 2:** Simulation Parameters.

Area Size	(3000 m × 3000 m × 1000 m)
Number of sensor nodes	400
simulation time	3000 s
Transmission range	500 m
Sensing range	250 m
Sensor Speed ($V_{is}$)	2.5 m/s
k	0, 1, 2, 3, 4, 5
Water currents speed ($V_{wc}$) based on the water depth	(0–200 m) 2 m/s
	(200–400 m) 1.7 m/s
	(400–600 m) 1.3 m/s
	(600–800 m) 0.9 m/s
	(800–1000 m) 0.5 m/s

## Data Availability

No data were used to support this study.

## Abbreviations

The following abbreviations are used in this manuscript:

UWSN	Underwater Sensor Network
3D-DVFA	3D Distributed Virtual Force Algorithm
SA-DVFA	Self-Adjustment Distributed Virtual Force Algorithm
